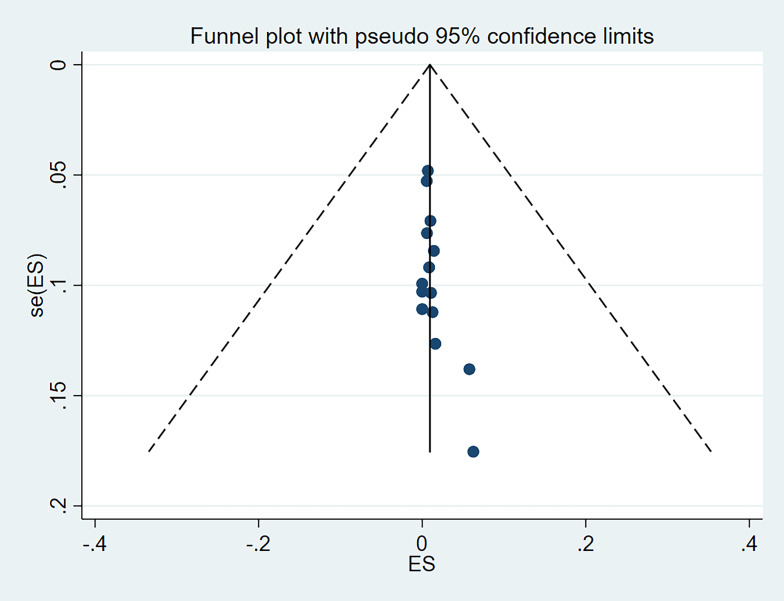# Frequency of lymph node metastases at different neck levels in patients with oral squamous cell carcinoma: a systematic review and meta-analysis

**DOI:** 10.1097/JS9.0000000000001953

**Published:** 2024-07-22

**Authors:** Yi-Fu Yu, Lei-Ming Cao, Zi-Zhan Li, Nian-Nian Zhong, Guang-Rui Wang, Yao Xiao, Qiu-Ji Wu, Bing Liu, Lin-Lin Bu

**Affiliations:** a State Key Laboratory of Oral and Maxillofacial Reconstruction and Regeneration, Key Laboratory of Oral Biomedicine Ministry of Education, Hubei Key Laboratory of Stomatology, School and Hospital of Stomatology, Wuhan University; b Department of Oral and Maxillofacial - Head Neck Oncology, School and Hospital of Stomatology, Wuhan University, Wuhan, People’s Republic of China; c Department of Radiation and Medical Oncology, Hubei Key Laboratory of Tumor Biological Behavior, Hubei Provincial Clinical Research Center for Cancer, Zhongnan Hospital of Wuhan University, Wuhan, China

**Keywords:** lymph node metastasis, neck dissection, oral squamous cell carcinoma, precision surgery, systematic review

## Abstract

**Background:**

Currently, neck dissection is a standard treatment for the majority of oral squamous cell carcinoma (OSCC) patients. However, the procedure can lead to a series of complications, significantly reducing patient quality of life and even affecting the antitumor immune response in patients undergoing immunotherapy. Therefore, in the era of precision surgery, gaining a deeper understanding of the patterns of lymph node metastasis (LNM) in OSCC is crucial.

**Materials and methods:**

Literature searches were performed on PubMed, Embase, Web of Science, Cochrane Library, WANFANGDATA, and China National Knowledge Infrastructure (CNKI) (inception to 10 April 2024). In addition, a manual searching was conducted in Scopus, Google Scholar, and Education Resources Information Center (ERIC). The authors included observational studies that evaluated the frequency of LNM in OSCC patients. Systematic review and a random effects model meta-analysis were performed.

**Results:**

The search identified 4694 articles, of which 17 studies included in our study. The authors calculated the frequency of LNM according to the data reported in the articles. Frequency of LNM=number of patients with positive lymph node / number of patients with OSCC. The frequency of LNM was 12% in level I (95% CI: 0.11–0.15, *I* ^2^=38.01%), 20% in level II (95% CI: 0.17–0.22, *I* ^2^=47.71%), 10% in level III (95% CI: 0.08–0.12, *I* ^2^=49.10%), 2% in level IV (95% CI: 0.01–0.03, *I* ^2^=27.58%), 1% in level V (95% CI: 0.00–0.01, *I* ^2^=11.37%).

**Conclusion:**

The frequency of LNM is consistent with the ‘cascade theory’ and appears to be no significant difference from different primary sites. The frequency of LNM were low in levels I–III and were very low in level IV–V, which implicated that more conservative treatments may be considered for OSCC in the future. This study will help clinicians better determine the extent of surgery and preserve lymph nodes during neck dissection.

## Introduction

Highlights

- The frequency of lymph node metastases was 12% in level I, 20% in level II, 10% in level III, 2% in level IV, and 1% in level V.
- The frequency of lymph node metastases appears to be no significant difference from different primary sites.
- This study will help clinicians better determine the extent of surgery and preserve lymph nodes during neck dissection, according to the ‘less is more’ concept.

Oral cancer is the sixth most common cancer in the world, globally accounting for 377 713 new cases and 177 757 deaths in 2020, representing an increase in new cases from 2018^1–5^. It is estimated that more than 90% of all oral cancer are oral squamous cell carcinoma (OSCC)^6^. In patients with OSCC, clinical evidence of lymph node metastasis (LNM) is observed in about 40%, while rates of occult metastasis vary between 15 and 34%^7–11^. Moreover, LNM is an important prognostic factor for a patient with OSCC, for the reason that the 5-year survival rate can decrease to below 20% when cervical metastasis occurs^12^.

Currently, neck dissection, a series of routine surgical procedures for removing cancer that has or potentially has spread to lymph nodes in the neck, is the preferred treatment for LNM in OSCC and serves as preventive measure against occult LNM^13^. Selective neck dissection is mainly employed for patients with an N0 neck status, focusing on the targeted removal of specific cervical lymph nodes (levels I, II, and III). However, in some instances, it is also applied to treat patients with N+ neck conditions^14–16^. For patients exhibiting N+ neck conditions, a modified radical neck dissection is frequently recommended to minimize the risk of recurrence^17^. Nonetheless, this procedure can result in postoperative complications, including shoulder dysfunction and disability, which may be temporary or, in some cases, permanent^18–21^. In addition, patient’s postoperative antitumor immune function may be compromised by neck dissection^22^. Based on a pivotal study published in Cell, lymph nodes play an important role in the antitumor immune responses, as the integrity of lymph nodes can affect the efficacy of immunotherapies^23^. Hence, it is essential to optimize the extent of neck dissection and minimize patient risk and subsequent morbidity. Preserving as many metastasis-free lymph nodes as possible cannot only enhance the patient’s quality of life but also boost the immune response to combat tumor recurrence. Furthermore, this approach supports the realization of the ‘3L’ goals in cancer treatment: living, living long, and living lively^24^. In order to optimize neck dissection and preserving the potential of immunotherapy, we need to first figure out the frequency of LNM.

Although, metastases to different neck levels are well demonstrated individually and several observational studies have been completed. Nonetheless, some of the existing studies have only explored LNM to level IIb, excluding level I, IIa, III, IV, V^17,25,26^. Some studies do not have separate data for each level^27,28^. Some studies have really small sample sizes^29–31^. Small sample sizes, single-center study, and different and complex statistical analysis methods have resulted in existing studies being of low scientific value, which preclude generalization of results and leads to ambiguity and uncertainty in clinical decision-making. In order to obtain a higher level of clinical evidence about the pattern of LNM in OSCC, we summarized the results of previous studies and preformed this systematic review and meta-analysis, hoping to provide some references for clinical decision-making in neck dissection surgery.

## Materials and methods

This systematic review was performed according to the Preferred Reporting Items for Systematic Reviews and Meta-Analyses (PRISMA, Supplemental Digital Content 1, http://links.lww.com/JS9/D121, Supplemental Digital Content 2, http://links.lww.com/JS9/D122) guidelines and A Measurement Tool to Assess Systematic Reviews 2 (AMSTAR2, Supplemental Digital Content 3, http://links.lww.com/JS9/D123)^32–34^. The protocol was registered on International Prospective Register of Systematic Reviews (PROSPERO).

### Eligibility criteria

Inclusion criteria were defined according to the PICOS questions: Participants: Patients who were pathological diagnosed as OSCC and whose primary sites were from the buccal mucosa or floor of mouth or oral tongue or alveolar ridge or retromolar trigone or hard palate^35^. Interventions: Neck dissection surgery. Comparison: Given that this study was a single-arm analysis, it didn’t include any comparisons. Outcomes: The primary interest was in assessing the frequency of LNM across various levels of the neck. The frequency of LNM was calculated according to the data reported in the articles. Frequency of LNM=number of patients with positive lymph node / number of patients with OSCC. The classification of neck levels follows the recommendations of the American Head and Neck Society^36^. Study: observational studies.

Exclusion criteria were defined as follows: (a) articles including patients undergoing tumor-related neoadjuvant chemotherapy or radiotherapy before surgery; (b) full-text not available in the English language or Chinese language; (c) articles consisting of animal studies, case reports, case series with fewer than 20 patients, review articles, conference abstracts, and duplicate publications; and (d) studies could not provide outcome data and could not convert the number of neck dissections or the number of positive lymph node into the number of patients with LNM.

If relevant studies included both patients with OSCC and patients with oropharynx squamous cell carcinoma, only the patients with OSCC were considered for analysis. When it was not possible to figure out the lymph node statue of patients who had bilateral neck dissection, only patients who had bilateral neck dissection were excluded.

### Information sources and search strategy

We searched the articles from the inception to 10 April 2024. Searches were conducted across four international electronic databases: PubMed, Embase, Web of Science, and the Cochrane Library. Key search terms were ‘oral squamous cell carcinoma’ AND ‘lymph node ratio’. For each concept, a mix of controlled vocabulary (MeSH and EMTREE) and text words was utilized in databases employing subject headings, while databases without subject headings primarily depended on text words. In order to ensure the comprehensiveness of this literature, two Chinese database was further searched, WANFANGDATA and China National Knowledge Infrastructure (CNKI). Key search terms were the synonyms of ‘oral squamous cell carcinoma’ AND ‘lymph node ratio’ in Chinese. Each concept used a combination of controlled vocabulary and text words in Chinese. Furthermore, a manual searching was conducted in Scopus, Google Scholar, and Education Resources Information Center (ERIC). References identified in relevant articles were screened for additional studies missed in the initial search. The search strategies are available in full detail in Supplementary Item 1 (Supplemental Digital Content 4, http://links.lww.com/JS9/D124).

### Selection process

After collecting all pertinent full-text articles, two authors carefully reviewed the reference lists of each selected paper to identify any studies that might have been missed. The search results were imported into EndNote 20 (Clarivate Analytics), where duplicates were subsequently eliminated. The screening process was conducted on EndNote 20 with titles and abstracts screened independently by two authors. Records lacking adequate details in their titles and abstracts were kept for further evaluation. Subsequently, these two authors separately assessed the full-text papers of each eligible paper. In the situation of disagreements regarding the inclusion of specific literature, a senior author was consulted to resolve the disagreements.

### Data collection process, data items, and effect measures

For each included article, two authors extracted all data into a preconstructed data table in Microsoft Excel. The following data was extracted: first author name, year of publication, sample size, number of patients with positive lymph node, number of patients with OSCC, neck dissection type, T-stage, N-stage, and primary site. The main outcomes were the number of patients with positive lymph node and the number of patients with OSCC. Based on the results of pathological testing of the specimens from the neck dissection surgery, the number of patients with positive lymph node in different levels was quantified, so as the number of patients with OSCC. According to the statistical methods of most studies, the frequency of LNM was the effect size. Frequency of LNM=number of patients with positive lymph node / number of patients with OSCC. Data needed for quantitative analyses were directly obtained from the original articles or derived from the original data. This step was performed independently by two authors, and any disagreements were resolved by discussing with a senior author.

**Figure 1:**
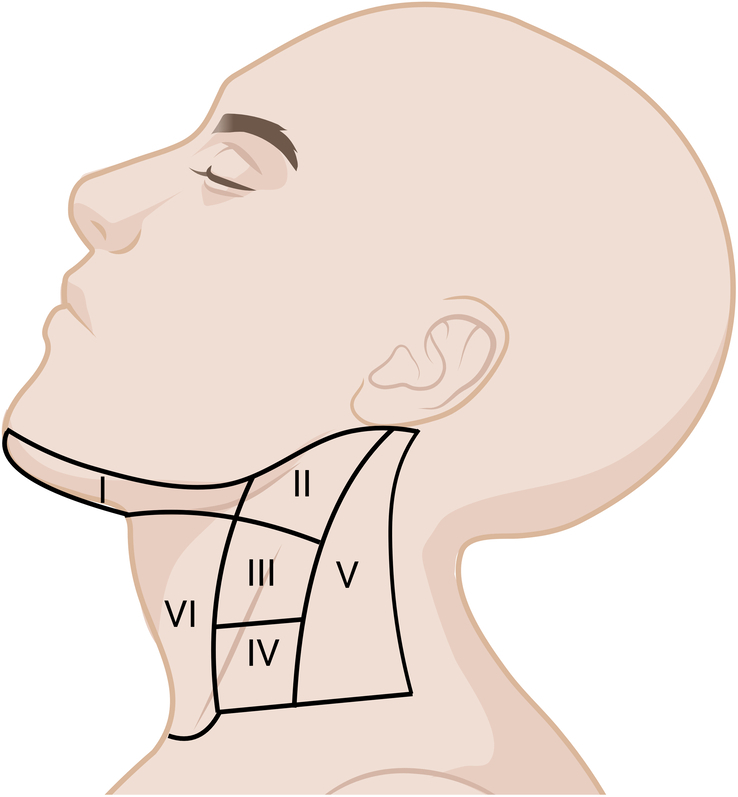
Schematic diagram of the neck levels.

The neck levels of lymph nodes follow the classification recommended by the American Head and Neck Society. Figure 1 provides a schematic representation of the neck level classification^37^.

### Risk of bias assessment

The quality of included articles were evaluated with the ‘Joanna Briggs Institute (JBI) critical appraisal tool’^38^. The JBI critical appraisal tool comprises 10 questions that focus on assessing the internal validity and risk of bias in case series studies, specifically targeting issues of confounding, selection, and information bias, while also emphasizing the significance of clear reporting^38^. For each study included, an assessment and scoring of ten items took place, providing the options ‘yes’, ‘no’, ‘unclear’, and ‘not applicable’ for responses. The overarching bias risk for every study was appraised according to a specific criteria: (a) a low bias risk was determined when all ten questions received a ‘yes’ response; (b) a high bias risk was designated if any question was answered with ‘no’; (c) an unclear bias risk was identified if any of the questions were marked as ‘unclear’^39^.

### Synthesis methods

This is a single arm meta-analysis. Statistical analysis was performed using STATA 15 (StataCorp LLC). The random-effects model was used to obtain pooled percentage estimates of level I–V nodal metastases. The heterogeneity among the effect sizes from individual studies was evaluated utilizing the *I*² index. This process involved the analysis of heterogeneity through the *I*² statistic, classifying it into low (25 to 50%), moderate (50 to 75%), or high (over 75%)^40^. Additionally, a sensitivity analysis was carried out to ascertain the robustness of the results.

### Reporting bias assessment

The potential publication bias for the outcomes were visually inspected using funnel plots and assessed by means of Begg and Mazumdar and Egger’s tests. A *P*-value more than 0.05 indicated no publication bias^41^.

### Certainty assessment

The certainty of evidence was assessed using the grading of recommendations, assessment, development, and evaluations tool (GRADE)^42,43^. The initial evidence of the observational studies was low and three factors could further enhance the certainty of evidence (dose–response, large effect, and plausible confounding); five factors could further decrease the certainty of evidence (inconsistency, risk of bias, imprecision, indirectness, and publication bias). Based on these criteria, the degree of certainty of the evidence was assessed and ultimately classified as very low, low, moderate, or high.

## Results

### Study selection

**Figure 2:**
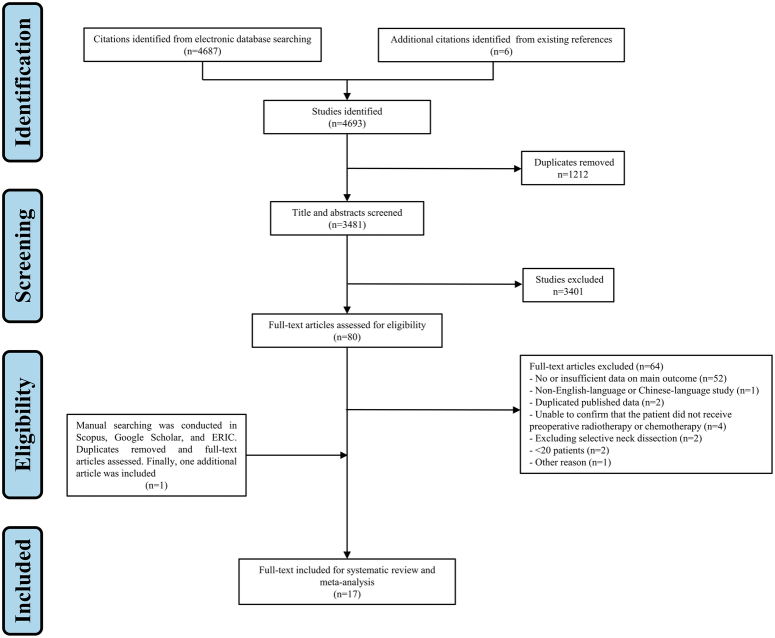
Study flow diagram.

In total, 4693 articles were identified through initial database searching, including 1298 articles from PubMed, 982 from Embase, 570 from Web of Science, 1216 from the Cochrane Library, 192 from WANFANGDATA, 429 from CNKI, and 6 additional articles were identified from existing references. After excluding 1212 articles that were duplicated, the remaining 3481 articles were searched by title and abstract. Following the exclusion of 3401 articles, the remaining 80 articles underwent a thorough full-text review and screening, as there was insufficient information in the title and abstract to assess whether they were eligible. Then, 64 articles were excluded for the following reasons: no or insufficient data on main outcome (*n*=52)^28,44–94^, non-English-language or Chinese-language study (*n*=1)^95^, duplicated published data (*n*=2)^96,97^, unable to confirm that the patient did not receive preoperative radiotherapy or chemotherapy (*n*=4)^98–101^, excluding selective neck dissection (*n*=2)^102,103^, <20 patients (*n*=2)^30,31^, and vague presentation (*n*=1)^104^. Woolgar’s study was excluded because it used a vague word ‘peppering’ to presenting the outcome without a clear definition^104^. In addition, manual searching was conducted in Scopus, Google Scholar, and ERIC. After duplicates removing and full-text articles assessing, one additional article was included for systematic review and meta-analysis^105^. Finally, a total of 17 records were included for single arm meta-analysis^105–121^. The PRISMA flow diagram of the study selection was shown in Figure 2.

### Study characteristics and results of individual studies

Seventeen studies describing a total of 2323 patients (range, 32–432 patients) were included for final analysis. All patients were pathologically diagnosed with OSCC who have undergone neck dissection surgery, including clinically negative (cN0) and cN+ cases in which no previous treatment had been administered for OSCC. The overall histological examination confirmed frequency of LNM was 13% in level I (256 of 1999, ranged between 7 and 21%), 20% in level II (394 of 1999, ranged between 13 and 35%), 10% in level III (193 of 1999, ranged between 6 and 21%), 2% in level IV (49 of 1999, ranged between 0 and 6%), 1% in level V (19 of 2013, ranged between 0 and 6%). The details of study characteristics are described in Table 1.

**Table 1:** Study characteristics of meta-analysis.

First author	Year	Case	Country	Study design	T stage	cN0/cN+	pN0/pN+	Primary site	Neck dissection type
Prakash Mishra^106^	2009	81	India	Prospective study	T1: 24 T2: 41 T3: 16	48/33	47/34	Tongue, Floor of mouth, Buccal mucosa, Lower alveolus, Lip up to vermilion border, Retromolar trigone	Modified radical neck dissection Supraomohyoid neck dissection Elective neck dissection Therapeutic neck dissection
J. A. Woolgar	2007	359	England	Retrospective study	T1: 54 T2: 116 T3: 24 T4: 162	Not stated	202/157	Cheek, Upper alveolus, Lower alveolus, Retromolar, Floor of mouth, Tongue	Radical neck dissection Modified radical neck dissection Selective neck dissection
Yu Oikawa^108^	2021	432	Japan	Retrospective study	Not stated	Not stated	321/111	Tongue	Not stated
KAZUYUKI KAINUMA^109^	2012	93	Japan	Retrospective study	Not stated	Not stated	39/54	Oral cavity	Superselective neck dissection
Naiboglu	2011	32	United States	Retrospective study	Not stated	Not stated	14/18	Oral cavity	Superselective neck dissection
Avi Khafif^111^	2001	51	United States	Prospective study	T1: 18 T2: 26 T3: 7	51/0	38/13	Tongue	Selective neck dissection
An-Kui Yang	2003	140	China	Retrospective study	T1, T2, T4	140/0	104/36	Tongue	Selective neck dissection
Xuan Zhang^113^	2008	52	China	Prospective study	T1: 13 T2: 30 T3: 7 T4: 2	Not stated	26/26	Tongue	Modified radical neck dissection Supraomohyoid neck dissection
Bao-Yuan Ren	2002	118	China	Retrospective study	T1: 33 T2: 52 T3: 24 T4: 9	118/0	85/33	Tongue	Not stated
Xiu-Wen Luan	2005	94	China	Retrospective study	T1: 21 T2: 40 T3: 23 T4: 10	94/0	55/39	Tongue	Radical neck dissection Modified radical neck dissection
Dong Wu	2022	102	China	Prospective study	T1+T2: 90 T3:12	102/0	69/33	Cheek, Tongue, Gingiva, Mandible, Soft palate	Supraomohyoid neck dissection
Qi-Lin Gong	2016	101	China	Retrospective study	T1: 64 T2: 37	101/0	79/22	Tongue	Functional neck dissection
Kai-Liu Wu^118^	2014	171	China	Retrospective study	T1: 65 T2: 106	171/0	131/40	Tongue	Supraomohyoid neck dissection Radical neck dissections
Fu-Yong Sui	2020	62	China	Retrospective study	T1: 27 T2: 35	Not stated	38/24	Tongue	Not stated
Zhu-Ming Guo	2002	79	China	Retrospective study	T1: 20 T2: 32 T3: 15 T4: 12	39/40	58/21	Floor of mouth	Radical neck dissection Elective neck dissection Supraomohyoid neck dissection Submandibular triangular dissection
Li-Shan Wang	2018	157	China	Retrospective study	T1: 49 T2: 108	157/0	109/48	Tongue	Radical neck dissection Modified radical neck dissection Expanded supraomohyoid neck dissection (I–IV)
Sriharsha Haranadh^105^	2018	199	India	Prospective study	T1: 51 T2: 84 T3: 14 T4: 50	114/85	125/74	Buccal mucosa, Tongue, Retromolar trigone, Lower alveolus, Lip, Floor of mouth	Modified neck dissection Selective neck dissection

Supraomohyoid neck dissection (dissection of level I, II, III nodes); Expanded supraomohyoid neck dissection (dissection of level I, II, III, IV nodes); Radical neck dissection (dissection of level I, II, III, IV, V nodes); Modified radical neck dissection (dissection of level I, II, III, IV, V nodes); Functional neck dissection (dissection of level I, II, III, IV, V nodes); Submandibular triangular dissection (dissection of level I, II nodes); Elective neck dissection (dissection of level I, II, III nodes); Superselective neck dissection (dissection of level I, II, III nodes); Selective neck dissection (dissection of level I, II, III nodes).

T stage: Unit is the number of patients; cN0/cN+: Unit is the number of patients; pN0/pN+: Unit is the number of patients.

### Risk of bias in studies

Quality assessment risk of bias was evaluated using the ‘JBI critical appraisal tool’ which contains 10 questions that relate to the internal validity and risk of bias of case series designs, particularly confounding, selection, and information bias, in addition to the importance of clear reporting^38^. Details of risk of bias of each study was shown in Table 2. Two study did not have clear reporting of the demographics of the participants and was considered as having high risk of bias^106,111^. Apart from this, the risk of bias for all studies was concentrated on the questionable completeness of the included cases. Most articles either did not have complete inclusion of participants or did not describe this detail specifically and were considered as having a high risk of bias or unclear risk of bias^106–113,116–121^.

**Table 2:** Quality assessment of included studies.

Study	1	2	3	4	5	6	7	8	9	10	Risk of bias
Sriharsha Haranadh 2018^105^	YES	YES	YES	YES	YES	YES	YES	YES	Not applicable	YES	Low
Prakash Mishra 2009^106^	YES	YES	YES	YES	NO	NO	YES	YES	Not applicable	YES	High
J. A. Woolgar 2007	YES	YES	YES	YES	Unclear	YES	YES	YES	Not applicable	YES	Unclear
Yu Oikawa 2021^108^	YES	YES	YES	YES	NO	YES	YES	YES	Not applicable	YES	High
KAZUYUKI KAINUMA 2012^109^	YES	YES	YES	YES	Unclear	YES	YES	YES	Not applicable	YES	Unclear
Naiboglu 2011	YES	YES	YES	YES	Unclear	YES	YES	YES	Not applicable	YES	Unclear
Avi Khafif 2001^111^	YES	YES	YES	YES	Unclear	NO	YES	YES	Not applicable	YES	High
An-Kui Yang 2003	YES	YES	YES	YES	Unclear	YES	YES	YES	Not applicable	YES	Unclear
Xuan Zhang 2008^113^	YES	YES	YES	YES	Unclear	YES	YES	YES	Not applicable	YES	Unclear
Bao-Yuan Ren 2002	YES	YES	YES	YES	YES	YES	YES	YES	Not applicable	YES	Low
Xiu-Wen Luan 2005	YES	YES	YES	YES	YES	YES	YES	YES	Not applicable	YES	Low
Dong Wu 2022	YES	YES	YES	YES	Unclear	YES	YES	YES	Not applicable	YES	Unclear
Qi-Lin Gong 2016	YES	YES	YES	YES	Unclear	YES	YES	YES	Not applicable	YES	Unclear
Kai-Liu Wu 2014^118^	YES	YES	YES	YES	Unclear	YES	YES	YES	Not applicable	YES	Unclear
Fu-Yong Sui 2020	YES	YES	YES	YES	Unclear	YES	YES	YES	Not applicable	YES	Unclear
Zhu-Ming Guo 2002	YES	YES	YES	YES	Unclear	YES	YES	YES	Not applicable	YES	Unclear
Li-Shan Wang 2018	YES	YES	YES	YES	Unclear	YES	YES	YES	Not applicable	YES	Unclear

Questions: 1. Were there clear criteria for inclusion in the case series? 2. Was the condition measured in a standard, reliable way for all participants included in the case series? 3. Were valid methods used for identification of the condition for all participants included in the case series? 4. Did the case series have consecutive inclusion of participants? 5. Did the case series have complete inclusion of participants? 6. Was there clear reporting of the demographics of the participants in the study? 7. Was there clear reporting of clinical information of the participants? 8. Were the outcomes or follow-up results of cases clearly reported? 9. Was there clear reporting of the presenting sites’/clinics’ demographic information? 10. Was statistical analysis appropriate?

#### Results of syntheses

**Figure 3:**
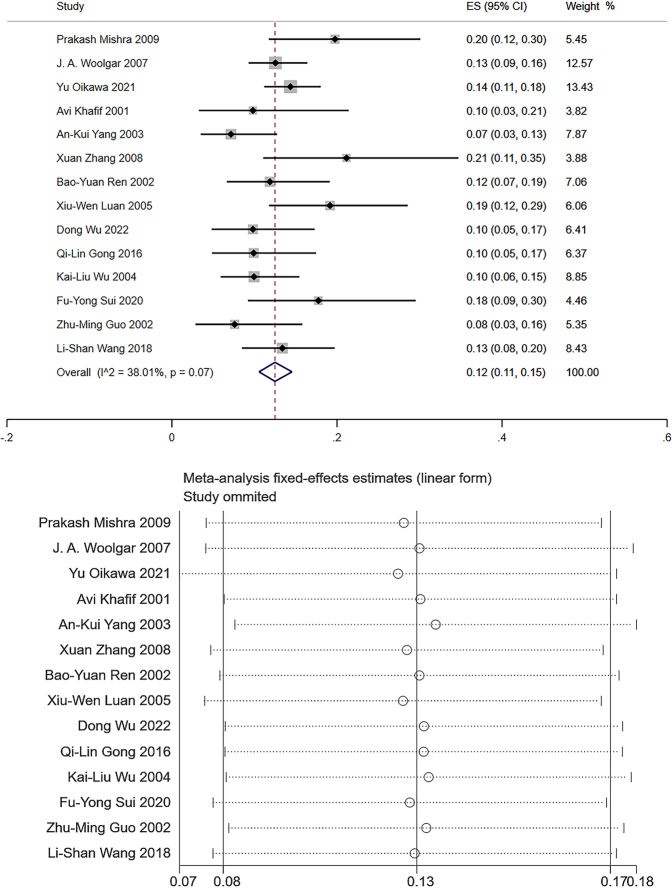
The frequency of lymph node metastasis (LNM) and sensitivity analysis for frequency of LNM at level I. The pooled mean frequency of LNM was 12% in level I (95% CI: 0.11–0.15, *I* ^2^=38.01%).

Level I: Data from 14 studies which measured the frequency of level I LNM were pooled to give a total of 1999 patients for statistical analysis^106–108,111–121^. The pooled mean frequency of LNM was 12% in level I (95% CI: 0.11–0.15, *I* ^2^=38.01%, Fig. 3). The heterogeneity was defined as low. Given the differences in TNM staging for patients and primary site in the original study, this level of heterogeneity was acceptable. Sensitivity analysis had been performed, and the graph showed that the results of meta-analysis were basically stable (Fig. 3).

**Figure 4:**
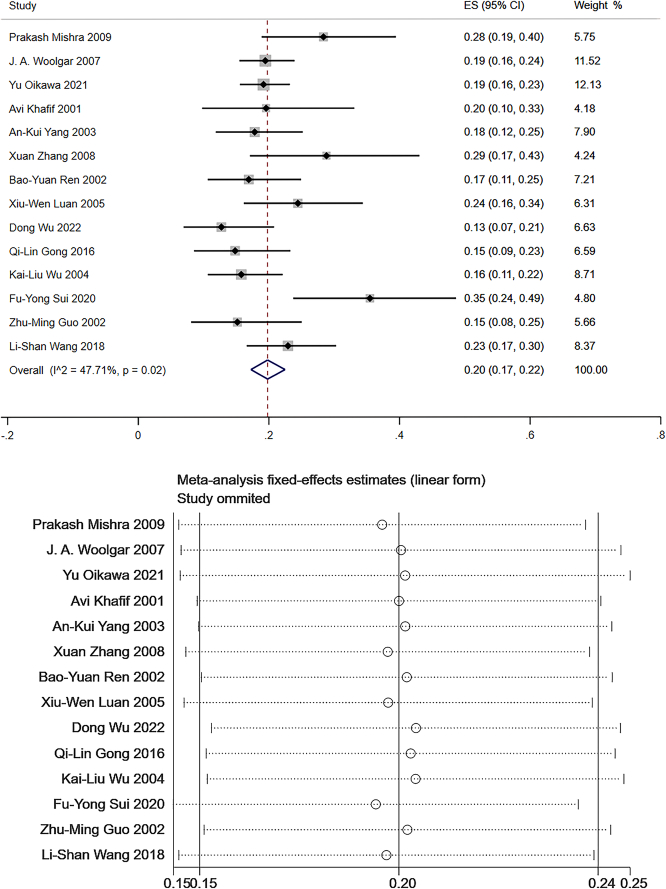
The frequency of lymph node metastasis (LNM) and sensitivity analysis for frequency of LNM at level II. The pooled mean frequency of LNM was 20% in level II (95% CI: 0.17–0.22, *I* ^2^=47.71%).

Level II: Data from 14 studies which measured the frequency of level II LNM were pooled to give a total of 1999 patients for statistical analysis^106–108,111–121^. The pooled mean frequency of LNM was 20% in level II (95% CI: 0.17–0.22, *I* ^2^=47.71%, Fig. 4). The heterogeneity was low. A sensitivity analysis was conducted, and the resulting graph indicated that the findings of the meta-analysis were fundamentally stable (Fig. 4).

**Figure 5:**
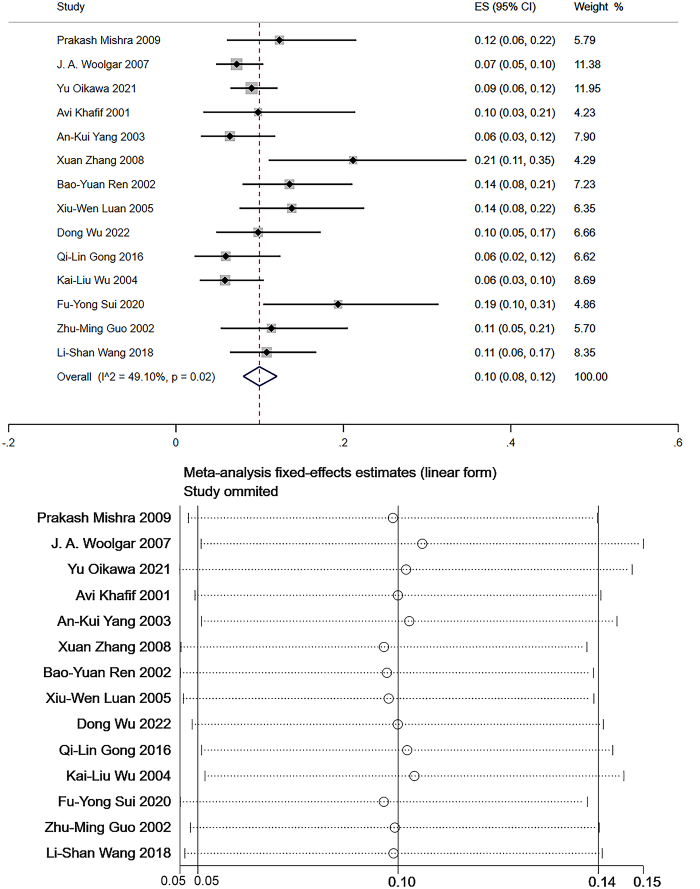
The frequency of lymph node metastasis (LNM) and sensitivity analysis for frequency of LNM at level III. The pooled mean frequency of LNM was 10% in level III (95% CI: 0.08–0.12, *I* ^2^=49.10%).

Level III: Data from 14 studies which measured the frequency of level III LNM were pooled to give a total of 1999 patients for statistical analysis^106–108,111–121^. The pooled mean frequency of LNM was 10% in level III with low heterogeneity (95% CI: 0.08–0.12, *I* ^2^=49.10%, Fig. 5). Sensitivity analysis showed that the robustness of the results was decent (Fig. 5).

**Figure 6:**
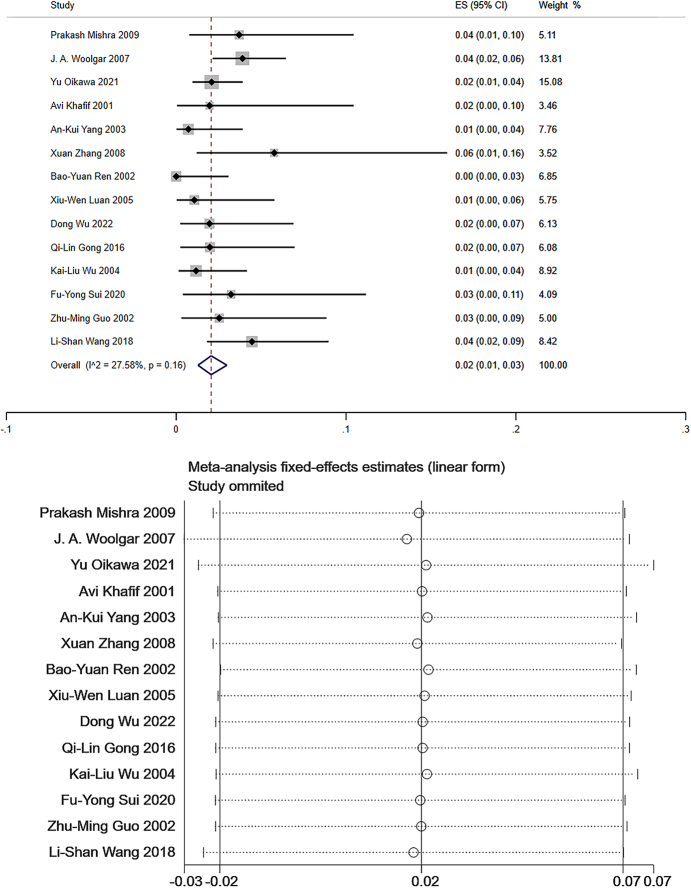
The frequency of lymph node metastasis (LNM) and sensitivity analysis for frequency of LNM at level IV. The pooled mean frequency of LNM was 2% in level IV (95% CI: 0.01–0.03, *I* ^2^=27.58%).

Level IV: Data from 14 studies which measured the frequency of level IV LNM were pooled to give a total of 1999 patients for statistical analysis^106–108,111–121^. The pooled mean frequency of LNM was 2% in level IV (95% CI: 0.01–0.03, *I* ^2^=27.58%, Fig. 6). The *I* ^2^ statistic indicated low heterogeneity. Sensitivity analysis had been performed, and the graph showed that the results of meta-analysis were basically stable (Fig. 6).

**Figure 7:**
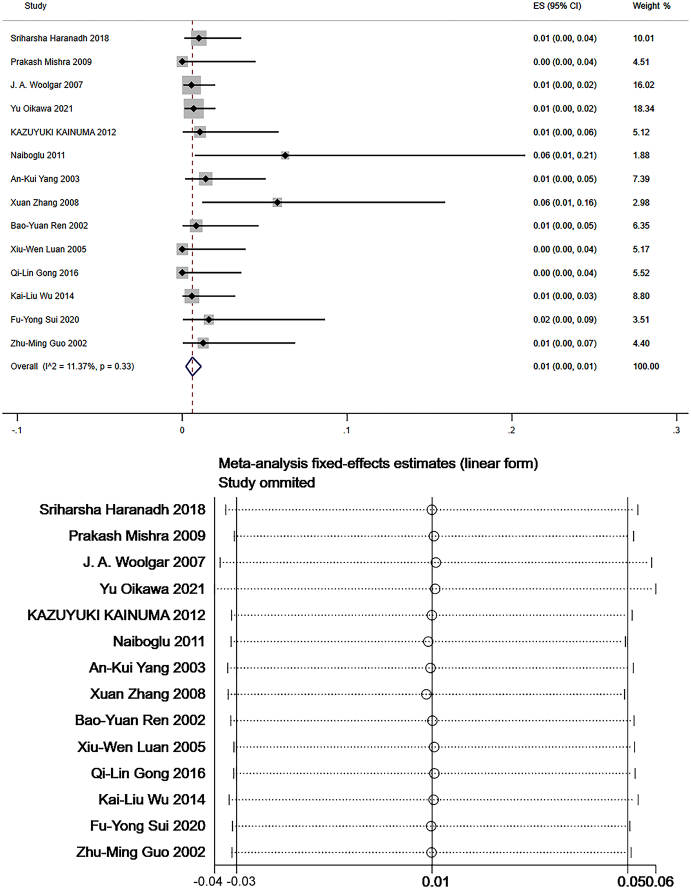
The frequency of lymph node metastasis (LNM) and sensitivity analysis for frequency of LNM at level V. The pooled mean frequency of LNM was 1% in level V (95% CI: 0.00–0.01, *I* ^2^=11.37%).

Level V: Data from 14 studies which measured the frequency of level V LNM were pooled to give a total of 2013 patients for statistical analysis^105–110,112–115,117–120^. The pooled mean frequency of LNM was 1% in level V (95% CI: 0.00–0.01, *I* ^2^=11.37%, Fig. 7). There was no heterogeneity. Sensitivity analysis showed that the results of meta-analysis were stable (Fig. 7).

### Reporting biases

Begg and Mazumdar and Egger’s tests were conducted and showed no significant risk of publication bias in level I (*P*=0.584 and *P*=0.787, respectively), level II (*P*=0.228 and *P*=0.291, respectively), level IV (*P*=0.381 and *P*=0.873, respectively), risk of publication bias in level III (*P*=0.029 and *P*=0.025, respectively), and level V (*P*=0.049 and *P*=0.043, respectively). The funnel plot was visually assessed and no outliers were found (Supplementary Item 2-6, Supplemental Digital Content 5, http://links.lww.com/JS9/D125, Supplemental Digital Content 6, http://links.lww.com/JS9/D126, Supplemental Digital Content 7, http://links.lww.com/JS9/D127, Supplemental Digital Content 8, http://links.lww.com/JS9/D128, Supplemental Digital Content 9, http://links.lww.com/JS9/D129).

### Certainty of evidence

The GRADE tool was used to assess the certainty of evidence. A low certainty of evidence was identified for the frequency of level I–V for the reason that all of the included studies were observational studies. The specific information regarding the certainty of the evidence was presented in Supplementary Item 7 (Supplemental Digital Content 10, http://links.lww.com/JS9/D130).

## Discussion

### Interpretation

In this study, the frequency of LNM is 12% in level I, 20% in level II, 10% in level III, 2% in level IV, 1% in level V. The frequency of LNM is consistent with the ‘cascade theory’, which indicates that OSCC LNM should first appear in proximate levels I, II, and III lymph nodes, with fewer metastases in level IV and V^122^. Compared with a previous study whose frequency of LNM was 15.58% in level I, 12.54% in level II, 1.29% in level III, 0% in level IV, this result showed slightly lower frequency of LNM in level I, slightly higher frequency of LNM in level IV and conspicuous higher frequency of LNM in level II and level III^123^. The different may be caused by the reason that most of the patients in Sangeet *et al*.’s study had buccal mucosa squamous cell carcinoma compared to tongue squamous cell carcinoma in this study. Unfortunately, the specific frequency of LNM could not be compared quantitatively in a uniform manner with some other large sample size studies, due to differences in statistical methods, but the ordering of the frequency of LNM at different neck levels was comparable. In a prospective study of 583 neck dissections, the order of the frequency of LNM from highest to lowest was level II, level I, level III, level IV, level V in tongue site, which was consistent with the results of this study. Moreover, the order of the frequency of LNM from highest to lowest was level I, level II, level III, level V, level IV in buccal mucosa site, which was highly similar to the results of Sangeet *et al*.’s study^68,123^. This reflected the potential influence of primary site on frequency of LNM at different neck levels.

However, contrary to views expressed above, there appeared to be no significant difference in frequency of LNM from different primary sites in this study^68,107,124–126^. Our study included patients from different primary sites, such as tongue, floor of mouth, buccal mucosa, lower alveolus, lip up to vermilion border, retromolar trigone, gingiva, mandible, and cheek, but the results of the pooled analysis did not have significant heterogeneity and have good stability. This finding is similar to the results of Gouri *et al*.’s study, as the order of the frequency of LNM from highest to lowest was level I, level II, level III from all primary site besides the tongue and the frequency of LNM in level IV, and level V were significantly lower than that in level I, level II, and level III from all primary sites^68^. This was also consistent with the findings of Woolgar’s study^104^. Nevertheless, this finding required further research, considering the majority of the primary site is tongue. Notably, this study did not take the frequency of LNM in sublevels such as IIb into account, which may be influenced by different primary sites seriously. Clinicians should exercise caution when adopting this conclusion and rigorously evaluate it.

### Limitations of evidence and review processes

Notably, several concerns remain to be considered. Existing studies report the status of LNM in different ways. Some studies use different delineation of the neck lymph node levels such as the Japan Neck Dissection Study Group classification^127,128^. Some studies used the number of patients to indicate the status of LNM, some used the number of lymph nodes, some used the number of neck dissections, and others used the lymph node ratio^59,68,104,129^. Some studies considered bilateral neck dissection, while some did not^68,107^. These inconsistencies in reporting and statistical methods make it very difficult to perform secondary research and increased unknown risk of bias between different studies. Considering that LNM is an extremely critical indicator in clinical cancer research, it is urgently recommended that a reporting standard should be designed for this important clinical issue.

Furthermore, statistical publication bias was showed in level III and V. However, this was a single-arm meta-analysis and does not involve negative or positive outcomes. The results reported in the included studies are objective observations of disease characteristics, so despite the fact that publication bias was statistical showed in level III and V, we assumed that researchers’ preference for publishing positive results does not affect our results. Due to the inclusion of observational studies, the certainty of evidence was rated as low initially. Nevertheless, the results did not have any factors that could seriously reduce the certainty of evidence, which indicated that the evidence remained credible. However, several factors in the GRADE tool were not suitable for this study, including the publication bias, which represented a limitation of this study.This study also has some other limitations. First, each study had its own unique inclusion and exclusion criteria and the ratio of cN0/cN+ was not consistent for each study. Second, most of the included researches reported on tongue squamous cell carcinoma. Regarding primary sites other than the tongue, their limited representation in research findings could contribute to observed heterogeneity. Additionally, this meta-analysis, which relied on aggregated data from published literature rather than individual patient data from each study, may introduce further inaccuracies. This is particularly relevant for patients who underwent bilateral neck dissection, as it might affect the precision of the results. Fourth, due to the long-time spans of the included studies, the accuracy of reported LNM in level I–V comes into question, as the anatomical boundaries of certain neck levels had been redefined in 2002 and modified in 2008^36,37^. Fifth, due to the limitations of the original research, we are unable to conduct further subgroup analyses based on the size of the primary sites. As the reader reads this study, the results should be rigorously evaluated, considering the effects of the heterogeneity described above.

### Implications for practice and future research

At present, neck dissection is essential for most patients with OSCC. Nonetheless, Bree *et al*.^130^ review indicates that about 70–80% of patients with cT1-2N0 OSCC have no cancer present in the cervical lymphatics according to the final pathology after undergoing selective neck dissection. In accordance with our findings, the frequency of LNM was highest in level II, relatively low in level I, III, and very low in level IV, V. Lymph nodes have an indispensable position in antitumor immune response. After immunotherapy, progenitor exhausted CD8+ T cells underwent differentiation into intermediate-exhausted CD8+ T cells (Tex-int) within uninvolved lymph nodes. Subsequently, Tex-int entered the bloodstream and underwent an expansion in the blood, before trafficking to infiltrate in tumors.

The objective of immunotherapy was to initiate antitumor immune response, primarily by activating the lymph nodes^131^. However, the dissection of lymph nodes prior to treatment resulted in the removal of essential areas where T cells survived and could be activated. Maintaining the integrity of lymph nodes until the completion of immunotherapy and allowing for the activation of the immune response within the lymph nodes during immunotherapy could enhance the efficacy of immunotherapy against solid tumors and further promote a positive response. It was undeniable that removing the positive lymph nodes was important, as OSCC could disseminate via tumor-draining lymph nodes and lymphatic vessels. However, removing these lymph nodes before immunotherapy might affect the T cell responses within lymph nodes, which could potentially compromise the effectiveness of immunotherapy. This demonstrated the essential and paradoxical role of lymph nodes in OSCC^132^. If an effective immune response could be initiated prior to the neck dissection surgery, more T cells would be retained in the body to against OSCC and prevent it from recurrence^23^. Furthermore, the preservation of lymph nodes could enhance patients’ quality of life, which was consistent the 3L goals—living, live long, and live lively—that we have proposed^24^.

The most recent study has demonstrated that neoadjuvant chemoimmunotherapy is a safe and efficacious treatment option with prognosis bona and high response rates for locally advanced resectable OSCC^133^. Notably, the neoadjuvant chemoimmunotherapy treatment strategy has showed a promising major pathological response for locally advanced OSCC, as well as an improvement in overall survival^134^. Our clinical case has also demonstrated that tumor immunotherapy can lead to complete disappearance of metastatic cancer cells in lymph nodes^24^. Furthermore, expanded lymph node dissection has been demonstrated to reduce the efficacy of immunotherapy, contrary to the conventional belief that ‘more is better’ in lymph node dissection^135^. Combined with lymph nodes preservation, the potential of immunotherapy remained largely untapped.

This inspired us to consider whether we could optimize current surgical guidelines for patients who have received immunotherapy, in order to routinely preserve lymph nodes in level IV and V. Preserving the lymph nodes can improve the effectiveness of immunotherapy and the more effective immunotherapy can further reduce the extent of surgery and the risk of distant metastatic in turn^23,136^. Considering that the highest frequency of LNM was just 20% in level II, for patients with cN0 lymph node in level I–III, it may be possible to further optimize surgical guidelines by considering watching and waiting rather than direct surgical removal. According to the ‘less is more’ concept which means that neck dissection surgery can bring better functional and esthetic results with fewer and smaller incisions, we hope this study will help clinicians better determine the extent of surgery and preserve lymph nodes during neck dissection^137^.

In the future, more clinical researches are needed to advance the development of precision surgery. There are still many unanswered questions. For instance, the design of individualized surgical plan for patients who have received immunotherapy and patients who have received other treatments, such as radiotherapy. Further investigations are required to comprehend the patterns of LNM at different neck levels from different OSCC primary sites. Additionally, the correlation between different primary sites and LNM in different sublevels should be investigated too. Notably, the relationship between TNM staging, imaging results and frequency of LNM needs to be further investigated in conjunction with imaging-related data.

## Conclusion

In conclusion, the findings of this systematic review and meta-analysis suggest that the frequency of LNM was 12% in level I, 20% in level II, 10% in level III, 2% in level IV, 1% in level V and there appears to be no significant difference in frequency of LNM from different primary sites. Further research needs to be conducted to figure out the LNM status of patients who have received immunotherapy and further modified the extent of neck dissection. Clinicians are advised to approach the findings of this review with caution, given that the methodological and clinical diversity among the included studies cannot be disregarded.

## Ethic approval

Not applicable.

## Consent

Not applicable.

## Source of funding

This study was supported by the Fundamental Research Funds for the Central Universities (Wuhan University, Clinical Medicine + X, 2042024YXB017), Postdoctoral Science Foundation of China (2018M630883 and 2019T120688), Hubei Province Chinese Medicine Research Project (ZY2023Q015), Natural Science Foundation of Hubei Province (2023AFB665), Medical Young Talents Program of Hubei Province, Wuhan Young Medical Talents Training Project to L.-L. Bu. and Youth Interdisciplinary Special Fund of Zhongnan Hospital of Wuhan University (ZNQNJC2022003). The study sponsors had no involvement.

## Author contribution

Y.-F.Y.: investigation, methodology, conceptualization, writing – original draft, visualization, writing – review and editing; L.-M.C.: methodology, conceptualization, writing – original draft, writing – review and editing; Z.-Z.L. and N.-N.Z.: investigation, methodology, conceptualization, writing – review and editing; G.-R.W.: investigation, methodology, and conceptualization; Y.X.: writing – review and editing and conceptualization; Q.-J.W.: conceptualization, methodology, writing – review and editing, supervision, and funding acquisition; B.L.: supervision, writing – review and editing, and project administration; L.-L.B.: conceptualization, supervision, project administration, funding acquisition, writing – review and editing. All authors have reviewed and approved the final version of this manuscript for publication. Each author agrees to be accountable for all aspects of the work in ensuring that questions related to the accuracy or integrity of any part of the work are appropriately investigated and resolved.

## Conflicts of interest disclosure

The authors declare that they have no known competing financial interests or personal relationships that could have appeared to influence the work reported in this paper.

## Research registration unique identifying number (UIN)

PROSPERO: CRD42023475459.

## Guarantor

Lin-Lin Bu.

## Data availability statement

The journal requires authors to include in any articles that report results derived from research data to include a Data Availability Statement. Please confirm if any datasets generated during and/or analyzed during the current study are publicly available, available upon reasonable request, or if data sharing is not applicable to this article. Data sharing is applicable to this article.

## Provenance and peer review

This paper is not invited.

## Presentation

Not applicable.

## Supplementary Material

**Figure:**
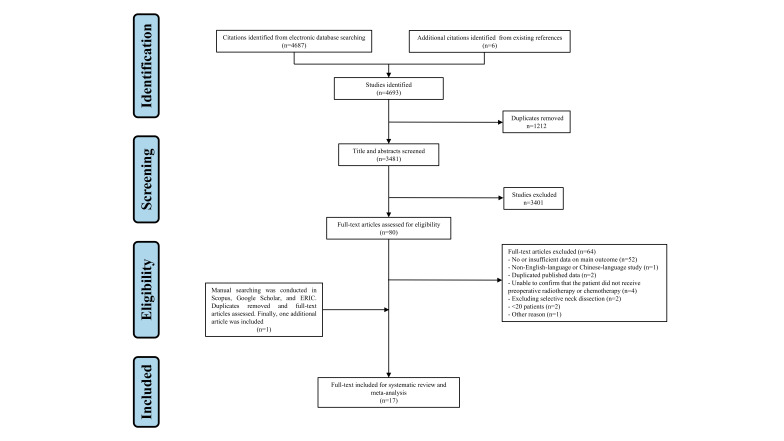


**Figure:**
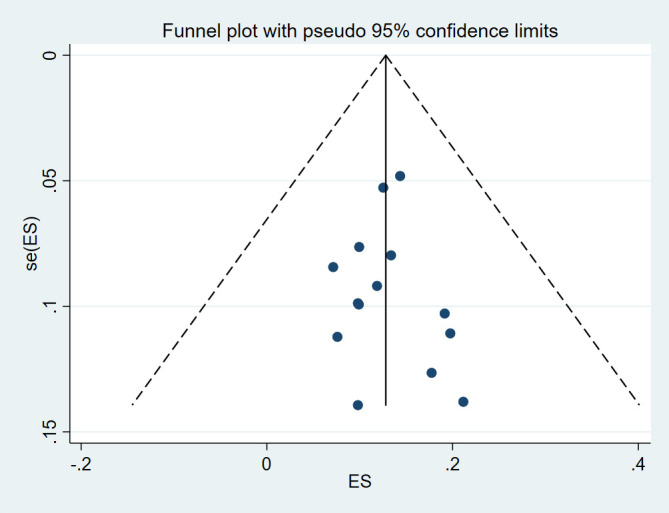


**Figure:**
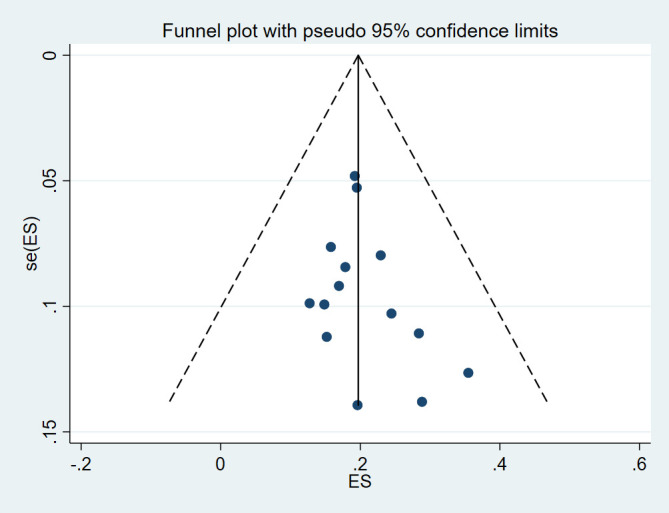


**Figure:**
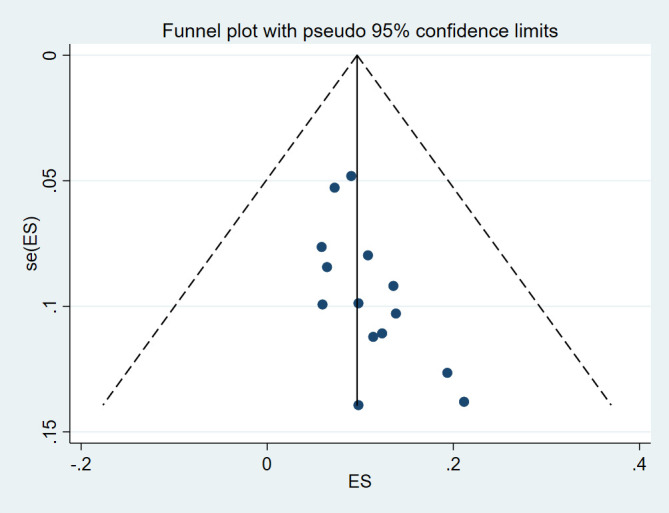


**Figure:**
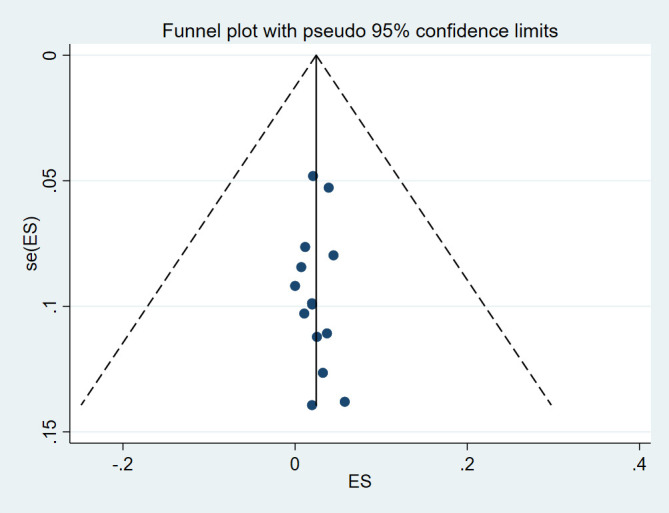


**Figure:**