# Supplementary material for: Frequency of lymph node metastases at different neck levels in patients with oral squamous cell carcinoma: a systematic review and meta-analysis
Source: Int J Surg. 2024 Jul 22;111(1):1285–300. doi: 10.1097/JS9.0000000000001953 (PMC11745673; doi:10.1097/JS9.0000000000001953)
Supplement: Supplementary file 3 [file js9-111-1285-s004.docx]

1. **Data extraction for meta-analysis**

| Study | number of patients with metastases | number of all patients |
| --- | --- | --- |
| Prakash Mishra 2009 | 16 | 81 |
| J. A. Woolgar 2007 | 45 | 359 |
| Yu Oikawa 2021 | 62 | 432 |
| Avi Khafif 2001 | 5 | 51 |
| An-Kui Yang 2003 | 10 | 140 |
| Xuan Zhang 2008 | 11 | 52 |
| Bao-Yuan Ren 2002 | 14 | 118 |
| Xiu-Wen Luan 2005 | 18 | 94 |
| Dong Wu 2022 | 10 | 102 |
| Qi-Lin Gong 2016 | 10 | 101 |
| Kai-Liu Wu 2014 | 17 | 171 |
| Fu-Yong Sui 2020 | 11 | 62 |
| Zhu-Ming Guo 2002 | 6 | 79 |
| Li-Shan Wang 2018 | 21 | 157 |

Level I data extraction

| Study | number of patients with metastases | number of all patients |
| --- | --- | --- |
| Prakash Mishra 2009 | 23 | 81 |
| J. A. Woolgar 2007 | 70 | 359 |
| Yu Oikawa 2021 | 83 | 432 |
| Avi Khafif 2001 | 10 | 51 |
| An-Kui Yang 2003 | 25 | 140 |
| Xuan Zhang 2008 | 15 | 52 |
| Bao-Yuan Ren 2002 | 20 | 118 |
| Xiu-Wen Luan 2005 | 23 | 94 |
| Dong Wu 2022 | 13 | 102 |
| Qi-Lin Gong 2016 | 15 | 101 |
| Kai-Liu Wu 2014 | 27 | 171 |
| Fu-Yong Sui 2020 | 22 | 62 |
| Zhu-Ming Guo 2002 | 12 | 79 |
| Li-Shan Wang 2018 | 36 | 157 |

Level II data extraction

| Study | number of patients with metastases | number of all patients |
| --- | --- | --- |
| Prakash Mishra 2009 | 10 | 81 |
| J. A. Woolgar 2007 | 26 | 359 |
| Yu Oikawa 2021 | 39 | 432 |
| Avi Khafif 2001 | 5 | 51 |
| An-Kui Yang 2003 | 9 | 140 |
| Xuan Zhang 2008 | 11 | 52 |
| Bao-Yuan Ren 2002 | 16 | 118 |
| Xiu-Wen Luan 2005 | 13 | 94 |
| Dong Wu 2022 | 10 | 102 |
| Qi-Lin Gong 2016 | 6 | 101 |
| Kai-Liu Wu 2014 | 10 | 171 |
| Fu-Yong Sui 2020 | 12 | 62 |
| Zhu-Ming Guo 2002 | 9 | 79 |
| Li-Shan Wang 2018 | 17 | 157 |

Level III data extraction

| Study | number of patients with metastases | number of all patients |
| --- | --- | --- |
| Prakash Mishra 2009 | 3 | 81 |
| J. A. Woolgar 2007 | 14 | 359 |
| Yu Oikawa 2021 | 9 | 432 |
| Avi Khafif 2001 | 1 | 51 |
| An-Kui Yang 2003 | 1 | 140 |
| Xuan Zhang 2008 | 3 | 52 |
| Bao-Yuan Ren 2002 | 0 | 118 |
| Xiu-Wen Luan 2005 | 1 | 94 |
| Dong Wu 2022 | 2 | 102 |
| Qi-Lin Gong 2016 | 2 | 101 |
| Kai-Liu Wu 2014 | 2 | 171 |
| Fu-Yong Sui 2020 | 2 | 62 |
| Zhu-Ming Guo 2002 | 2 | 79 |
| Li-Shan Wang 2018 | 7 | 157 |

Level IV data extraction

| Study | number of patients with metastases | number of all patients |
| --- | --- | --- |
| Sriharsha Haranadh 2018 | 2 | 199 |
| Prakash Mishra 2009 | 0 | 81 |
| J. A. Woolgar 2007 | 2 | 359 |
| Yu Oikawa 2021 | 3 | 432 |
| KAZUYUKI KAINUMA 2012 | 1 | 93 |
| Naiboglu 2011 | 2 | 32 |
| An-Kui Yang 2003 | 2 | 140 |
| Xuan Zhang 2008 | 3 | 52 |
| Bao-Yuan Ren 2002 | 1 | 118 |
| Xiu-Wen Luan 2005 | 0 | 94 |
| Qi-Lin Gong 2016 | 0 | 101 |
| Kai-Liu Wu 2014 | 1 | 171 |
| Fu-Yong Sui 2020 | 1 | 62 |
| Zhu-Ming Guo 2002 | 1 | 79 |

Level V data extraction

1. **Search query for Pubmed**

((((((((((("Head and Neck Neoplasms"[Mesh]) OR (Head[Title/Abstract] AND Neck Cancer[Title/Abstract])) OR (((((("Squamous Cell Carcinoma of Head and Neck"[Mesh]) OR (HNSCC[Title/Abstract])) OR (Oral Tongue Squamous Cell Carcinoma[Title/Abstract])) OR (Oral Squamous Cell Carcinoma[Title/Abstract])) OR (Oral Cavity Squamous Cell Carcinoma[Title/Abstract])) OR (Squamous Cell Carcinoma of the Mouth[Title/Abstract]))) OR (((((("Mouth Neoplasms"[Mesh]) OR (Mouth Cancer[Title/Abstract])) OR (Oral Neoplasm[Title/Abstract])) OR (Oral Cancer[Title/Abstract])) OR (Oral Cavity Cancer[Title/Abstract])) OR (Oral Cavity Neoplasms[Title/Abstract]))) OR (((("Gingival Neoplasms"[Mesh]) ) OR (Gingival Squamous Cell Carcinomas[Title/Abstract])) OR (Gingival Cancer[Title/Abstract]))) OR ((("Lip Neoplasms"[Mesh]) OR (Lip Squamous Cell Carcinomas[Title/Abstract])) OR (Lip Cancer[Title/Abstract]))) OR ((("Tongue Neoplasms"[Mesh]) OR (Tongue Squamous Cell Carcinomas[Title/Abstract])) OR (Tongue Cancer[Title/Abstract]))) OR ((("Jaw Neoplasms"[Mesh]) OR (Jaw Squamous Cell Carcinomas[Title/Abstract])) OR (Jaw Cancer[Title/Abstract]))) OR ((("Maxillary Neoplasms"[Mesh]) OR (Maxillary Squamous Cell Carcinomas[Title/Abstract])) OR (Maxillary Cancer[Title/Abstract]))) OR ((("Palatal Neoplasms"[Mesh]) OR (Palatal Squamous Cell Carcinomas[Title/Abstract])) OR (Palatal Cancer[Title/Abstract]))) OR ((((((((((((((((((((Retromolar Trigone Squamous Cell Carcinomas[Title/Abstract]) OR (Retromolar Trigone Cancer[Title/Abstract])) OR (Alveolus Squamous Cell Carcinomas[Title/Abstract])) OR (Alveolus cancer[Title/Abstract])) OR (Mouth Floor Squamous Cell Carcinomas[Title/Abstract])) OR (Mouth Floor Cancer[Title/Abstract])) OR (Mouth Mucosa Squamous Cell Carcinomas[Title/Abstract])) OR (Mouth Mucosa Cancer[Title/Abstract])) OR (Oral Mucosa Squamous Cell Carcinomas[Title/Abstract])) OR (Oral Mucosa Cancer[Title/Abstract])) OR (Buccal Mucosa Squamous Cell Carcinomas[Title/Abstract])) OR (Buccal Mucosa Cancer[Title/Abstract])) OR (Cheek Squamous Cell Carcinomas[Title/Abstract])) OR (Cheek Cancer[Title/Abstract])) OR (Bucca Squamous Cell Carcinomas[Title/Abstract])) OR (Bucca Cancer[Title/Abstract])) OR (Mandible Squamous Cell Carcinomas[Title/Abstract])) OR (Mandible Cancer[Title/Abstract])) OR (Chin Squamous Cell Carcinomas[Title/Abstract])) OR (Chin Cancer[Title/Abstract]))) AND (((((((((((((("Lymph Node Ratio"[Mesh]) OR (Node Ratio, Lymph[Title/Abstract])) OR (Ratio, Lymph Node[Title/Abstract])) OR (Rate of metastases[Title/Abstract])) OR (Prevalence of metastases[Title/Abstract])) OR (pattern of lymph node metastasis[Title/Abstract])) OR (Pattern of metastatic spread[Title/Abstract])) OR (Routes of metastatic spread[Title/Abstract])) OR (Distributions of lymph node metastases[Title/Abstract])) OR (Metastatic patterns[Title/Abstract])) OR (Lymph node status[Title/Abstract])) OR (Node Involvement Ratio[Title/Abstract]) ) OR (Metastatic Lymph Node Burden[Title/Abstract])) OR (number of positive lymph nodes[Title/Abstract]))
