# Supplementary material for: Frequency of lymph node metastases at different neck levels in patients with oral squamous cell carcinoma: a systematic review and meta-analysis
Source: Int J Surg. 2024 Jul 22;111(1):1285–300. doi: 10.1097/JS9.0000000000001953 (PMC11745673; doi:10.1097/JS9.0000000000001953)
Supplement: Supplementary file 4 [file js9-111-1285-s010.docx]

**Supplementary Item 7. GRADE tool for outcomes certainty assessment.**

| **Certainty assessment** | | | | | | | **№ of patients** | | **Results** | | **Certainty** |
| --- | --- | --- | --- | --- | --- | --- | --- | --- | --- | --- | --- |
| **№ of studies** | **Study design** | **Risk of bias** | **Inconsistency** | **Indirectness** | **Imprecision** | **Other considerations** | **With LNM** | **Overall number of patients** | **Pooled mean frequency of LNM** | **95% CI** |  |
| 14 | observational studies | not serious | not serious | not serious | not serious | none | 256 | 1999 | 0.12 | 0.11 to 0.15 | ⨁⨁◯◯ Low |
| 14 | observational studies | not serious | not serious | not serious | not serious | none | 394 | 1999 | 0.20 | 0.17 to 0.22 | ⨁⨁◯◯ Low |
| 14 | observational studies | not serious | not serious | not serious | not serious | none | 193 | 1999 | 0.10 | 0.08 to 0.12 | ⨁⨁◯◯ Low |
| 14 | observational studies | not serious | not serious | not serious | not serious | none | 49 | 1999 | 0.02 | 0.01 to 0.03 | ⨁⨁◯◯ Low |
| 14 | observational studies | not serious | not serious | not serious | not serious | none | 19 | 2013 | 0.01 | 0.00 to 0.01 | ⨁⨁◯◯ Low |

**CI:** confidence interval
